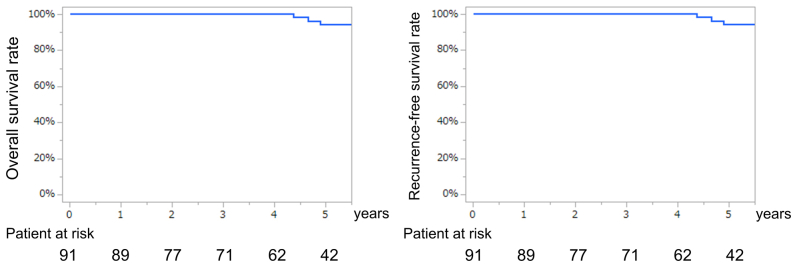# Radiologic Parameters Predicting the Histologic Invasiveness of Pure Ground-Glass Nodules

**DOI:** 10.1016/j.atssr.2024.02.009

**Published:** 2024-03-19

**Authors:** Yasuto Kondo, Masashi Mikubo, Masaaki Ichinoe, Shoko Hayashi, Dai Sonoda, Masahito Naito, Yoshio Matsui, Kazu Shiomi, Yukitoshi Satoh

**Affiliations:** 1 Department of Thoracic Surgery, Kitasato University School of Medicine, Sagamihara, Kanagawa, Japan; 2 Department of Thoracic Surgery, Kitasato University Medical Center, Kitamoto, Saitama, Japan; 3 Department of Pathology, Kitasato University School of Medicine, Sagamihara, Kanagawa, Japan

## Abstract

**Background:**

This study aimed to investigate the diagnostic performance of combined computed tomography (CT) and fluorine-18 fluorodeoxyglucose (FDG) positron emission tomography (PET) for predicting histologic invasiveness of pure ground-glass nodules (pGGNs).

**Methods:**

The study analyzed 91 patients who underwent resection of pGGNs and examined the correlation of pathologic invasiveness with preoperative CT and FDG PET findings.

**Results:**

Overall, 24, 36, and 31 patients had adenocarcinoma in situ (AIS), minimally invasive adenocarcinoma (MIA), and invasive adenocarcinoma (IAD), respectively. Compared with AIS and MIA, IAD was significantly correlated with larger CT size (*P* = .001), maximum CT value (*P* = .026), and high maximum standardized uptake value (SUVmax; *P* < .001). Multivariable logistic analyses revealed that CT size (odds ratio [OR], 3.848; *P* = .019) and SUVmax (OR, 4.968; *P* = .009) were independent predictors of histologic invasiveness. Receiver operating characteristic curve analysis revealed that a cutoff CT size value of 18 mm predicted histologic invasiveness with a sensitivity and specificity of 65% and 80%, respectively; similarly, a cutoff SUVmax value of 1.5 predicted histologic invasiveness with a sensitivity and specificity of 61% and 90%, respectively. Of 20 lesions with CT size ≥18 mm and SUVmax ≥1.5, 16 (80%) were IAD. Of 54 lesions with CT size <18 mm and SUVmax <1.5, 46 (85%) were non-IAD lesions. Furthermore, all pGGNs with SUVmax ≥2.5 were IAD.

**Conclusions:**

CT size and SUVmax were significantly correlated with the histologic invasiveness of pGGNs. These factors may aid in determining optimal surgical procedures.

## Visual Abstract

**Figure:**
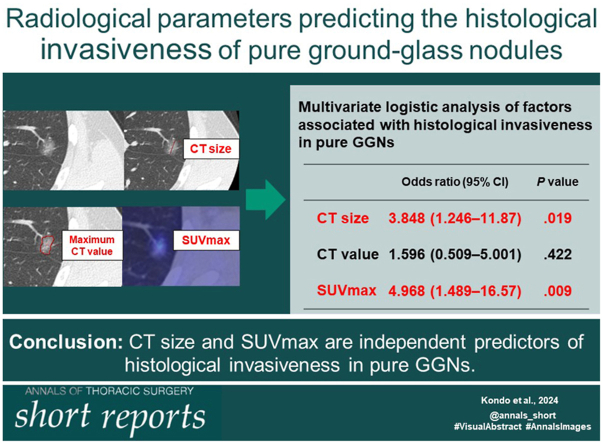

**In Short**

▪ Among pure ground-glass nodules (pGGNs), invasive adenocarcinoma (IAD) was significantly correlated with larger size on computed tomography (CT) and high maximum standardized uptake value (SUVmax).
▪ The cutoff CT size and SUVmax values for predicting histologic invasiveness were set at 18 mm and 1.5, respectively.
▪ Overall, 80% of the pGGNs with CT size ≥18 mm and SUVmax ≥1.5 were IAD; conversely, 85% of the pGGNs with CT size <18 mm and SUVmax <1.5 were non-IAD lesions.

The introduction of computed tomography (CT) for lung cancer screening has increased the probability of encountering pulmonary ground-glass nodules (GGNs) in clinical practice. Most pure GGNs (pGGNs; with ground-glass components and without solid components) histologically represent adenocarcinoma in situ (AIS) or minimally invasive adenocarcinoma (MIA), which are considered indolent types of adenocarcinoma. Sublobar resection has recently emerged as a successful treatment modality for GGN-dominant lung adenocarcinomas, thus making limited resection the standard procedure for pGGNs.^1^

The indications for limited resection are based on CT findings; therefore, precise assessment of CT images is crucial for surgical decision making. However, there may be discrepancies between radiographic findings and pathologic diagnoses, with 15% to 44% of the pGGNs turning out to be invasive adenocarcinoma (IAD).^2^, ^3^, ^4^, ^5^ To avoid undertreatment by performing limited resection of these lesions, predictors of the histologic invasiveness of pGGNs must be identified. Several studies have suggested that tumor size, CT value, and pleural indentation can predict histologic invasiveness; however, these findings remain contentious.^2^, ^3^, ^4^, ^5^

Fluorine-18 fluorodeoxyglucose (FDG) positron emission tomography (PET) with CT (PET-CT) is widely used to determine treatment strategies for non-small cell lung cancer.^6^^,^^7^ However, the role of PET-CT in predicting the histologic invasiveness of pGGNs has not been evaluated, and its diagnostic value remains unknown. Therefore, this study aimed to assess the diagnostic performance of combined CT and PET-CT and identify the clinicoradiologic features predictive of the histologic invasiveness of pGGNs.

## Patients and Methods

**Supplementary Figure 1:**
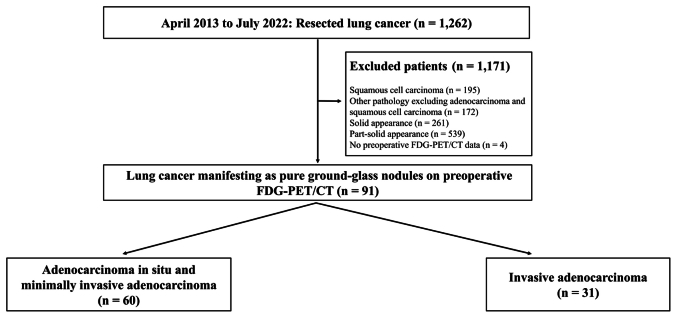


This study was approved by Kitasato University Hospital (Sagamihara, Kanagawa, Japan) Institutional Review Board (approval number: B21-274), and the requirement of informed consent was waived. This retrospective case control study, conducted at Kitasato University Hospital, focused on patients meeting the following criteria: primary lung cancer treated by surgical resection at Kitasato University Hospital between April 2013 and July 2022, primary lung adenocarcinoma confirmed on definitive pathologic examination, the presence of radiologically diagnosed pGGNs, and the availability of preoperative thin-slice CT and FDG PET-CT images. Collected data included patient characteristics, such as age, sex, smoking history (pack-years), CT tumor size, maximum CT values, maximum standardized uptake value (SUVmax), and the presence of pleural tags. On the basis of the histologic diagnosis, patients with pGGNs were categorized into AIS-MIA and IAD groups. The patient selection flow chart is shown in Supplemental Figure 1. The classification of AIS, MIA, and IAD was based on the World Health Organization classification system (fifth edition).^8^ Further methodologic details are available in the Supplemental Methods section.

## Results

**Supplementary Figure 2:**
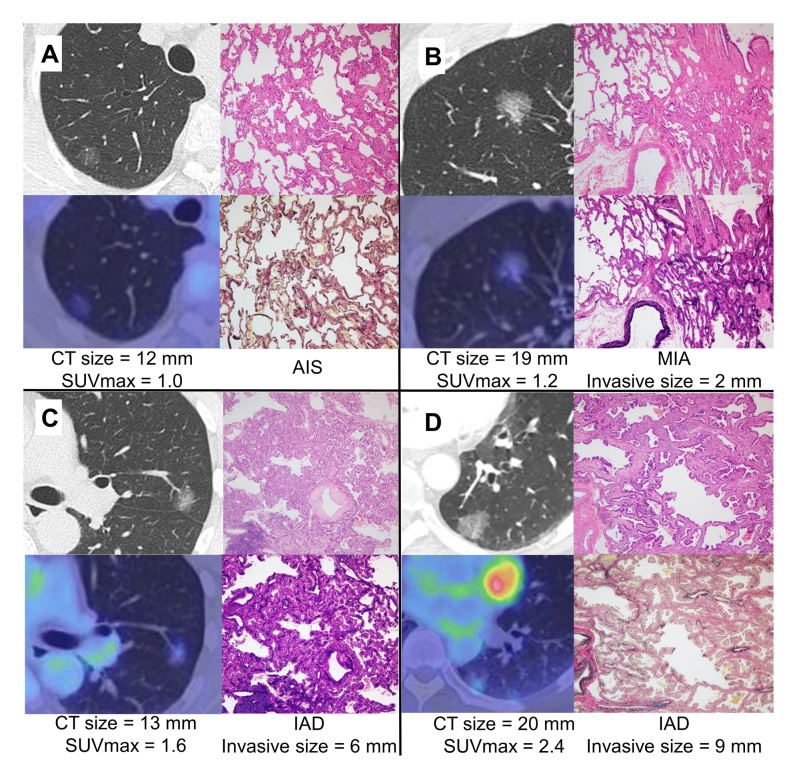


Of the 91 patients included in this study, 24 (26%), 36 (40%), and 31 (34%) had histologic diagnoses of AIS, MIA, and IAD, respectively. Representative imaging and pathologic findings of the pGGNs are presented in Supplemental Figure 2. The predominant histologic IAD patterns were lepidic (n = 12), papillary (n = 16), acinar (n = 1), solid (n = 1), and invasive mucinous (n = 1). Furthermore, 28 (32%), 25 (27%), and 38 (41%) patients underwent lobectomy, segmentectomy, and wedge resection, respectively. The reasons for lobectomy were insufficient surgical margins for segmental or partial resection (n = 14), the presence of tumor in the inner one-third of the lung field (n = 10), or both (n = 4). The mean CT size was 15.5 ± 6.5 mm (range, 5-43 mm). The mean sizes of the invasive components in AIS-MIA and IAD were 2.0 ± 1.8 mm (range, 0-5 mm) and 9.0 ± 4.1 mm (range, 6-17 mm), respectively. The pathologic stages in 24, 57, and 10 patients were 0, IA1, and IA2, respectively. No lymph node dissection, hilar node dissection, and mediastinal node dissection were performed in 44, 14, and 33 patients, respectively. Lymph node metastasis or lymphovascular invasion was not observed in any lesion. The median follow-up time was 4.8 years. The 5-year overall and relapse-free survival rates were both 94.2% (Supplemental Figure 3). No postoperative recurrence was observed.

The clinicoradiologic features of the AIS-MIA and IAD groups are shown in Table 1. The IAD group was significantly associated with larger CT size (*P* = .001), high maximum CT value (*P* =.026), high SUVmax (*P* < .001), and larger pathologic tumor diameter (*P* = .002).

**Table 1:** Patient Characteristics and Radiologic Features of Pure Ground-glass Nodules

Variable	AIS-MIA (n = 60)	IAD (n = 31)	*P* Value
Age, y	66 (57-75)	69 (64-74)	.243
Sex, male	23 (38.3)	17 (54.8)	.132
Smoking index, pack-years	12.9 ± 19.5	17.0 ± 21.6	.363
Pleural tag presence	23 (38.3)	16 (51.6)	.226
CT size, mm	13.8 ± 6.5	18.5 ± 5.3	.001
Maximum CT value, HU	−186 ± 155.2	−107 ± 166.3	.026
SUVmax	1.0 ± 0.4	1.6 ± 0.7	<.001
Pathologic size, mm	14.4 ± 6.3	19.5 ± 8.9	.002

Values are median (interquartile range), n (%), or mean ± SD.

AIS, adenocarcinoma in situ; CT, computed tomography; HU, Hounsfield units; IAD, invasive adenocarcinoma; MIA, minimally invasive adenocarcinoma; SUVmax, maximum standardized uptake value.

**Figure 1:**
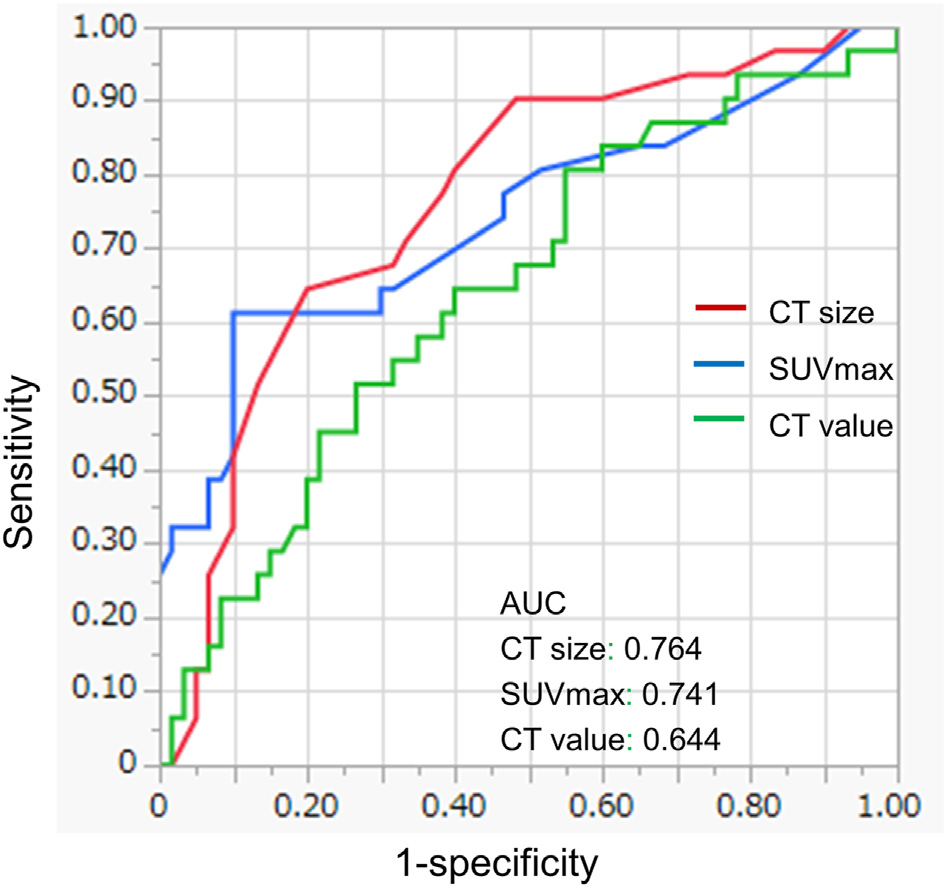
Receiver operating characteristic curves for histologic invasiveness predicted using computed tomography (CT) size (red), maximum standardized uptake value (SUVmax) (blue), and CT value (green). (AUC, area under the curve.)

Using receiver operating characteristic curve analysis, the maximum CT cutoff value for predicting histologic invasiveness was set at −232 Hounsfield units with an area under the curve (AUC) of 0.644 (Figure 1); the sensitivity, specificity, positive predictive value (PPV), and negative predictive value (NPV) were 80%, 45%, 42%, and 79%, respectively. Similarly, a CT size cutoff of 18 mm with an AUC of 0.764 yielded a sensitivity, specificity, PPV, and NPV of 65%, 80%, 65%, and 81%, respectively (Figure 1). SUVmax cutoff of 1.5 with an AUC of 0.741 yielded a sensitivity, specificity, PPV, and NPV of 61%, 90%, 72%, and 80%, respectively (Figure 1).

Table 2 presents the results of the analyses for predictors of histologic invasiveness. Univariate analysis revealed CT size (odds ratio [OR], 8.099; 95% CI, 3.026-21.67; *P* < .001), maximum CT value (OR, 2.805; 95% CI, 1.088-7.954; *P* = .032), and SUVmax (OR, 10.48; 95% CI, 3.620-30.35; *P* < .001) as significant factors. However, multivariable logistic analyses revealed only CT size (OR, 3.848; 95% CI, 1.246-11.87; *P* = .019) and SUVmax (OR, 4.968; 95% CI, 1.489-16.57; *P* = .009), but not maximum CT value (OR, 1.596; 95% CI, 0.509-5.001; *P* = .422), as independent predictors of histologic invasiveness.

**Table 2:** Factors Associated With Histologic Invasiveness of Pure Ground-glass Nodules

Variable	Univariate Analysis Odds Ratio (95% CI)	*P* Value	Multivariable Analysis Odds Ratio (95% CI)	*P* Value
Age, y	0.879 (0.329-2.346)	.798	…	…
Sex, male	1.953 (0.811-4.700)	.135	…	…
Smoking history, yes	1.099 (0.396-3.050)	.854	…	…
Pleural tag	1.715 (0.714-4.119)	.226	…	…
CT size	8.099 (3.026-21.67)	<.001	3.848 (1.246-11.87)	.019
Maximum CT value	2.805 (1.088-7.954)	.032	1.596 (0.509-5.001)	.422
SUVmax	10.48 (3.620-30.35)	<.001	4.968 (1.489-16.57)	.009

CT, computed tomography; SUVmax, maximum standardized uptake value.

We also examined clinicoradiologic predictors for histologic invasiveness in pGGNs ≤2 cm (typically considered for limited resection). In this cohort, the IAD group was significantly associated with larger CT size (*P* = .001) and high SUVmax (*P* < .001) (Supplemental Table 1). Univariate analysis revealed CT size (OR, 8.086; 95% CI, 2.126-30.76; *P* < .001) and SUVmax (OR, 13.75; 95% CI, 3.634-52.02; *P* < .001) as significant factors of histologic invasiveness. Furthermore, multivariable logistic analyses revealed CT size (OR, 4.444; 95% CI, 1.061-18.60; *P* = .041) and SUVmax (OR, 8.204; 95% CI, 2.025-33.23; *P* = .003) as independent predictors (Supplemental Table 2).

**Figure 2:**
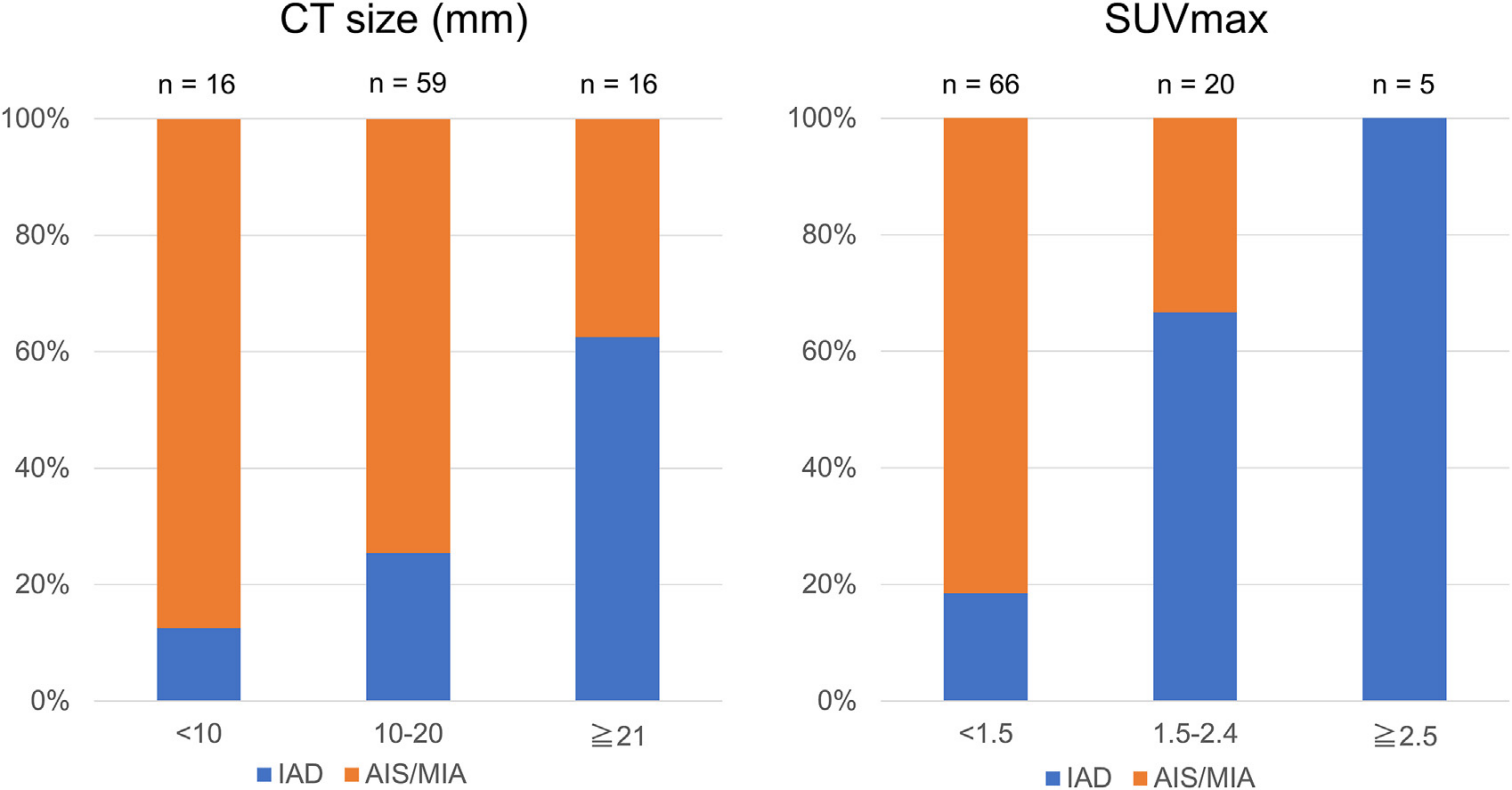
Correlation of computed tomography (CT) size and maximum standardized uptake value (SUVmax) with pathologic invasiveness of pure ground-glass nodules. (AIS/MIA, adenocarcinoma in situ or minimally invasive adenocarcinoma; IAD, invasive adenocarcinoma.)

The relationship between CT size and SUVmax and pathologic invasiveness in pGGNs is shown in Figure 2. Of the 25 patients with SUVmax ≥1.5, 18 (72%) had pathologic diagnoses of IAD. Notably, all patients with pGGNs with SUVmax ≥2.5 had IAD. Of the 75 patients with CT size ≤20 mm, 21 patients (28%) had IAD; of these, 11 (52%) exhibited SUVmax ≥1.5. Combining the CT size and SUVmax, 20 patients with CT size ≥18 mm and SUVmax ≥1.5 were identified; of these, 16 (80%) had IAD. Conversely, 54 patients with CT size <18 mm and SUVmax <1.5 were identified; of these, 46 (85%) had non-IAD lesions.

## Comment

Accurately assessing the risk of histologic invasiveness preoperatively is crucial. The discrepancies between radiographic findings and pathologic diagnoses can be attributed to the diversity of radiographic results and the complexity of their assessment. Previous studies revealed that CT size and the presence of solid components in GGNs exhibit measurement variability, which is especially significant for small and low-grade malignant tumors such as AIS and MIA.^9^ Given the challenge of consistent CT evaluation, combining modalities with CT may help predict the histologic invasiveness of GGN lesions.

Several studies have investigated the usefulness of SUVmax for predicting biologic malignancy in non-small cell lung cancer.^6^^,^^7^ Our study investigated the relationship between SUVmax and invasiveness of pGGNs. Our results indicate that SUVmax has a significant predictive performance for histologic invasiveness of pGGNs, either alone or in combination with CT size.

Recent developments in lung cancer surgery have led limited resection to become the standard for small peripheral lung cancers. A phase III study from the Japan Clinical Oncology Group (JCOG0804) focused on the suitability of sublobar resection for peripheral GGNs measuring ≤2 cm with a consolidation-to-tumor ratio of ≤0.25.^1^ Whereas more than one-half of the enrolled patients had a diagnosis of pGGNs, 7 patients received an unexpected diagnosis of IAD and required lobectomy or reoperation instead of limited resection. Although no SUVmax data in the JCOG0804 study are available, PET could have been useful in detecting such cases. In fact, our study demonstrated the detecting performance in the cohort with pGGNs ≤2 cm. Despite cost-effectiveness issues, obtaining PET results may be useful in cases where it is difficult to determine invasion on imaging. We hope that our results aid in identifying IAD manifesting as pGGNs and in selecting the best surgical approach.

This study has several limitations. It was a single-institutional, retrospective, case-control study. We could not provide data on optimal surgical procedures for pGGNs given the small sample size. Because only patients with surgically removed pGGNs that were pathologically proven to be adenocarcinomas were included, verification bias cannot be ruled out. A larger prospective study is needed to confirm our findings and explore ideal surgical approaches.

In conclusion, CT size and SUVmax were significantly correlated with the histologic invasiveness of pGGNs. Our results may help surgeons identify IAD manifesting as pGGNs and determine optimal surgical procedures.

## Figures and Tables

**Supplementary Figure 3:**