# Supplementary material for: Radiologic Parameters Predicting the Histologic Invasiveness of Pure Ground-Glass Nodules
Source: Ann Thorac Surg Short Rep. 2024 Mar 19;2(3):464–8. doi: 10.1016/j.atssr.2024.02.009 (PMC11708158; doi:10.1016/j.atssr.2024.02.009)
Supplement: Supplementary Figure Legend 1 [file mmc1.docx]

**Figure 1**

Patient flowchart.

CT: computed tomography; FDG-PET: ^18^F-fluorodeoxyglucose positron emission tomography
