# Supplementary material for: Radiologic Parameters Predicting the Histologic Invasiveness of Pure Ground-Glass Nodules
Source: Ann Thorac Surg Short Rep. 2024 Mar 19;2(3):464–8. doi: 10.1016/j.atssr.2024.02.009 (PMC11708158; doi:10.1016/j.atssr.2024.02.009)
Supplement: Supplementary Figure Legend 2 [file mmc2.docx]

**Figure 2**

Representative imaging and pathologic findings of pure GGNs. Axial CT image and photomicrographs of histology specimens with hematoxylin and eosin staining (×40) and Elastica van Gieson staining (×40).

(A) AIS, CT size = 12 mm and SUVmax = 1.0; (B) MIA, CT size = 19 mm and SUVmax = 1.2; (C) IAD, CT size = 13 mm and SUVmax = 1.6; (D) IAD, CT size = 20 mm and SUVmax = 2.4.

AIS, adenocarcinoma in situ; CT, computed tomography; GGN, ground-glass nodule; IAD, invasive adenocarcinoma; MIA, minimally invasive adenocarcinoma; SUVmax, maximum standardized uptake value.
