# Supplementary material for: Radiologic Parameters Predicting the Histologic Invasiveness of Pure Ground-Glass Nodules
Source: Ann Thorac Surg Short Rep. 2024 Mar 19;2(3):464–8. doi: 10.1016/j.atssr.2024.02.009 (PMC11708158; doi:10.1016/j.atssr.2024.02.009)
Supplement: Supplementary Figure Legend 3 [file mmc3.docx]

**Supplemental Figure 3**

The 5-year overall and recurrence-free survival rates of all patients.
