# Supplementary material for: Radiologic Parameters Predicting the Histologic Invasiveness of Pure Ground-Glass Nodules
Source: Ann Thorac Surg Short Rep. 2024 Mar 19;2(3):464–8. doi: 10.1016/j.atssr.2024.02.009 (PMC11708158; doi:10.1016/j.atssr.2024.02.009)
Supplement: Supplementary Methods [file mmc4.docx]

**Patients and methods**

***Surgical procedure***

Patients underwent wedge resection when the lesion was located in the outer third of the lung field; conversely, they underwent anatomic resection (such as lobectomy or segmentectomy) when the lesion was located in the inner third of the lung field or when multiple lesions were present in the same lobe. Lobectomy was performed when sufficient surgical margin could not be secured by segmental or partial resection.

***CT imaging features***

Helical computed tomography (CT) was performed with the patient in the supine position, and 1.25 mm high-resolution CT images of the lungs were obtained during a deep inspiratory breath hold using 64-detector row CT scanners (Optima 660; GE Healthcare, Tokyo, Japan). The scanning parameters were as follows: 512 × 512 matrices, 1.25 mm collimation, and a 0.5-second scan time at 120 kVp and 270 mA. The lung window level was 600 Hounsfield units (HU), and the window width was 1,500 HU. Pure GGNs were defined as shadows completely occupied by a ground-glass opacity with no solid regions. A lesion was classified as pure GGN following agreement between two authors experienced in reading chest CT scans (Y.K. and M.M.). The maximum CT value of each lesion was measured in areas excluding portions of apparent vessels, and the highest Hounsfield units were selected.

***^18^F-fluorodeoxyglucose (FDG)-positron emission tomography/computed tomography***

The imaging protocol used for most patients (n = 89) was as follows. The patients were instructed to fast for a minimum of 5 hours before an intravenous injection of 170–300 MBq ^18^F-fluorodeoxyglucose (FDG) and to relax for at least 1 hour before FDG-positron emission tomography (PET)/CT scanning. For imaging, Biograph mCT Flow® (Siemens Healthcare GmbH, Henkestr, Germany) with integrated 3-dimensional FDG-PET/CT scanners was used. Low-dose, non-enhanced CT images with a 2–4-mm section thickness for attenuation correction and localization of lesions identiﬁed by PET were obtained from the head to the pelvic ﬂoor in each patient following a standard protocol. Immediately after CT, PET covered the identical axial ﬁeld of view for 2–4 minutes per table position, depending on the patient’s condition and the scanner's performance. An iterative algorithm with CT-derived attenuation correction was used to reconstruct all PET images with a 50-cm ﬁeld of view. The imaging protocol for the other patients (n = 2) was almost the same, but Biograph TruePoint® (Siemens Healthcare GmbH, Henkestr, Germany) was used for imaging instead.

**Analysis of histopathological features**

All pathological specimens were formalin-fixed and stained with hematoxylin-eosin and Elastica van Gieson. The presence and size of the pathologically invasive component were evaluated by a lung pathologist (M.I.).

**Statistical analysis**

Categorical comparisons were performed using the Pearson chi-squared test for discrete data and the Student’s t-test or one-way analysis of variance for continuous data. The prediction accuracy of continuous variables for histological invasiveness was evaluated using a receiver operating characteristic (ROC) curve and the area under the curve (AUC). The cutoff value that yielded optimal sensitivity and specificity was determined using the Youden index. Prediction accuracy for pathological non-invasive cancer was expressed using sensitivity, specificity, positive predictive value (PPV), and negative predictive value (NPV). Logistic regression analysis was conducted to determine the association between clinical or radiographic variables and the histological invasiveness of pure GGNs. The potential factors associated with histological invasiveness were identified using univariate logistic regression analysis. To investigate whether SUVmax is useful for predicting histological invasiveness, we performed subgroup analyses in pure GGNs ≤2 cm. Factors with P-values <.05 in univariate analyses were included in multivariable regression models to identify the independent factors. Overall and recurrence-free survival rates were calculated from the date of surgery to the time of death and to the date of relapse or death from any cause, respectively, and were estimated using the Kaplan–Meier method.

All statistical analyses were performed using JMP 17 (SAS Institute, Cary, NC, USA). All tests were two-sided, and P-values <.05 were considered indicative of statistical significance.
