# Supplementary material for: Radiologic Parameters Predicting the Histologic Invasiveness of Pure Ground-Glass Nodules
Source: Ann Thorac Surg Short Rep. 2024 Mar 19;2(3):464–8. doi: 10.1016/j.atssr.2024.02.009 (PMC11708158; doi:10.1016/j.atssr.2024.02.009)
Supplement: Supplementary Table 1 [file mmc5.docx]

**Supplemental Table 1. Patient characteristics and radiological features of pure ground-glass nodules ≤2.0 cm**

| **Variable** | **AIS/MIA (n = 54)** | **IAD (n = 21)** | ***P* value** |
| --- | --- | --- | --- |
| Age (IQR) | 66 (42–83) | 67 (41–83) | .647 |
| Sex, male | 19 (35.1) | 11 (52.3) | .175 |
| Smoking index (pack-years) | 12.2 ± 19.0 | 18.0 ± 22.7 | .270 |
| Pleural tag (presence, %) | 20 (37.3) | 9 (42.8) | .643 |
| CT size (mm) | 12.2 ± 3.8 | 15.8 ± 3.6 | <.001 |
| Maximum CT value (HU) | -188 ± 156.4 | -119 ± 177.9 | .105 |
| SUVmax | 0.9 ± 0.3 | 1.4 ± 0.7 | <.001 |
| Pathological size (mm) | 13.4 ± 5.5 | 16.6 ± 7.9 | .051 |

AIS, adenocarcinoma in situ; CT, computed tomography; HU, Hounsfield units; IAD, invasive adenocarcinoma; IQR, interquartile range; MIA, minimally invasive adenocarcinoma; SUVmax, maximum standardized uptake value.
