# Supplementary material for: Radiologic Parameters Predicting the Histologic Invasiveness of Pure Ground-Glass Nodules
Source: Ann Thorac Surg Short Rep. 2024 Mar 19;2(3):464–8. doi: 10.1016/j.atssr.2024.02.009 (PMC11708158; doi:10.1016/j.atssr.2024.02.009)
Supplement: Supplementary Table 2 [file mmc6.docx]

**Supplemental Table 2. Factors associated with histological invasiveness of pure ground-glass nodules ≤2.0 cm**

| **Variable** | **Univariate analysis**  **Odds ratio (95% CI)** | ***P-*value** | **Multivariable analysis**  **Odds ratio (95% CI)** | **P-value** |
| --- | --- | --- | --- | --- |
| Age, years | 0.742 (0.232–2.371) | .614 |  |  |
| Sex, male | 2.026 (0.728–5.632) | .175 |  |  |
| Smoking history: yes | 1.391 (0.417–4.633) | .590 |  |  |
| Pleural tag | 1.275 (0.457–3.555) | .642 |  |  |
| CT size | 8.086 (2.126–30.76) | <.001 | 4.444 (1.061–18.60) | .041 |
| Maximum CT value | 2.553 (0.905–7.201) | .076 |  |  |
| SUVmax | 13.75 (3.634–52.02) | <.001 | 8.204 (2.025–33.23) | .003 |

CI, confidence interval; CT, computed tomography; SUVmax, maximum standardized uptake value.
